# Policy Knowledge and Abortion Access for US Active‐Duty Servicewomen: A Mixed‐Methods Study

**DOI:** 10.1111/jmwh.70049

**Published:** 2025-11-05

**Authors:** Caitlin Russell, Tiara Walz, Laura Manzo, Shelby Mueller, Sharon Messina, Sharon Arana, Keira Feng, Holly Harner

**Affiliations:** ^1^ University of Pennsylvania Philadelphia Pennsylvania; ^2^ US Army Medical Center of Excellence Ft. Sam Houston Texas; ^3^ Yale University Orange Connecticut; ^4^ University of Massachusetts – Boston Boston Massachusetts; ^5^ US Air Force Washington; ^6^ Jefferson College of Nursing Philadelphia Pennsylvania

**Keywords:** abortion, health equity, health policy, military and veterans, reproductive justice

## Abstract

**Introduction:**

More than 80,000 US military servicewomen are stationed in states enforcing abortion bans. US Department of Defense (DOD) policies must adhere to the Hyde Amendment, which restricts abortion coverage. Little research exists regarding active‐duty servicewomen's (ADSW) knowledge of these policies or their experiences accessing abortion care. This study examines the reproductive health policy knowledge and lived experiences of ADSW who have accessed abortion care.

**Methods:**

A 24‐item questionnaire was designed to measure reproductive health policy knowledge and explore reproductive health experiences among ADSW. Via secondary analysis, a subset of 178 participants self‐reported obtaining an abortion while on active duty. A convergent mixed‐methods design (quantitative and qualitative) was used.

**Results:**

Most participants (65%; n = 115) did not know TRICARE covered abortion costs in cases of rape or incest; 77% (n = 137) erroneously believed they required leadership permission to get an abortion; and 87% (n = 155) did not know they were entitled to convalescent leave to recover after an elective abortion. More than half (53%; n = 94) took personal leave to access abortion care; 46% (n = 82) traveled more than one hour; 48% experienced financial difficulties; 31% (n = 55) experienced negative professional repercussions; 16% (n = 29) received convalescent leave; 92% (n = 164) were not offered mental health counseling; and 77% (n = 137) felt they would have benefited from mental health counseling postabortion. Qualitative themes included a systemic lack of DOD abortion policy knowledge and lived experiences of accessing abortion care (eg, financial burdens, stigma).

**Discussion:**

Given the systemic lack of policy knowledge reported among study participants, the DOD should develop and implement a standardized abortion policy and access training for military health care professionals, leadership, and service members. Policies ensuring access to abortion should be adopted, codified, and implemented uniformly across all branches of service.

## INTRODUCTION

Ninety‐five percent of women serving in the US military are of reproductive age (18‐44).^1^ Active‐duty servicewomen (ADSW) experience higher rates of unplanned pregnancy compared with civilians, largely due to systemic barriers to accessing reliable contraception.^2^, ^3^ Furthermore, 24% of ADSW experience a sexual assault during their military service, which can result in an unplanned pregnancy, and among those who have deployed, they are more likely to experience a sexual assault and adverse pregnancy outcomes.^4^, ^5^ ADSW who are pregnant are placed on a *pregnancy profile*, which means they are not eligible for deployment, are potentially prohibited from performing certain job duties (eg, environmental exposures and physical risk), and may be unable to take part in training and assessments that impact future career prospects.^6^, ^7^ Placement on a nondeployable profile occurs regardless of intention to continue with the pregnancy. It was only recently (2022) that ADSW had the option to delay leadership notification of their pregnancy until 20 weeks’ gestation.^8^

**QUICK POINTS**

✦ Many US active‐duty servicewomen (ADSW) who have obtained abortion care are unfamiliar with the US Department of Defense policies addressing abortion coverage and access.
✦ A lack of knowledge among ADSW and a culture of stigmatization in the military lead to systemic barriers to care and personal or professional repercussions from other service members.
✦ Although 77% of study participants felt that postabortion mental health support would have been helpful, only 8% were offered such resources.
✦ There is an urgent need for the US Department of Defense to expand its efforts to educate military personnel on abortion‐related policies and ensure the rights and health care needs of ADSW are met.

There are currently more than 80,000 ADSW (more than 40%) stationed in states enforcing full or partial abortion bans.^9^ The majority of large US Department of Defense (DOD) facilities and training sites are located in states with abortion bans, meaning that ADSW will likely reside in these states at some point during their military career.^10^ Approximately 4100 ADSW seek abortion care annually, although this is likely underestimated due to social desirability bias.^9^, ^11^ The Hyde Amendment limits financial coverage for abortion services and restricts the use of military treatment facilities (MTF) for abortion care, prohibiting the DOD from funding or performing abortions except in cases of rape or incest or if the mother's life is endangered.^12^, ^13^ TRICARE, the DOD health care program, classifies abortion care that meets Hyde Amendment criteria as *covered* and all others as *noncovered* abortions.^14^ ADSW must seek care for noncovered abortions through nonmilitary providers and pay out of pocket for abortion services or abortion‐related complications (eg, infections, incomplete abortions).^14^ ADSWs are subject to DOD leave policies, which limit their freedom of movement compared with civilians. Military leadership retains full authority over when and where servicemembers can travel and may deny travel requests at their discretion.^15^ Such denials act as a barrier for ADSW seeking abortion care.^16^

ADSW also experience other significant barriers to abortion and postabortion care.^16^, ^17^, ^18^ These obstacles are caused by the complexity of navigating the military health care system to obtain abortion services^9^, ^19^ and broader sociocultural challenges within the military such as stigma, misinformation, and inadequate access to abortion resources and information.^18^, ^19^, ^20^ Unfortunately, the majority of previous studies did not examine abortion as the primary outcome of interest (eg, focusing on barriers to contraception access with abortion as an outcome), and this area of research remains underexplored.^11^ The Dobbs decision brings a renewed sense of urgency to abortion access research for this population, as the ruling compromises not only military readiness but also bodily autonomy and reproductive health care access for ADSW.^11^

Little research exists regarding ADSW's level of knowledge regarding the DOD policies governing access to abortion care (eg, coverage, convalescent leave, mental health support) or their experiences seeking abortion care. This gap in knowledge and access can lead to adverse health outcomes,^16^, ^20^, ^21^ as well as delayed care, financial burdens,^22^ and feelings of isolation and stigmatization that undermine unit cohesion and mission readiness.^9^, ^23^

Our study aims to address these critical gaps by (1) assessing policy knowledge of ADSW who have accessed abortion care and (2) exploring the lived experiences of ADSW who have accessed abortion care while in service. This research seeks to inform future studies and guide policymakers in developing strategies to promote equitable access to reproductive health care within the military and ensure ADSW are better supported in making informed decisions about their reproductive health.

## METHODS

This secondary analysis used a subset of participants (N = 178) from a larger data set exploring the experiences and policy knowledge of ADSW regarding reproductive health care. A convergent mixed‐methods design (quantitative and qualitative) allowed for more comprehensive and corroborated results, given the comparative nature of the research objectives.^24^ Inclusion criteria were (1) ADSW who (2) self‐reported having an elective abortion while serving on active duty and (3) completed the questionnaire. This study was granted exempt status by the University of Pennsylvania Institutional Review Board as it contained no identifiable data.

Participants were recruited through snowball sampling via email and closed social media groups, which required validation of active‐duty status. A collective of ADSW collaborated with academic partners to perform community‐engaged research exploring DOD reproductive health care policy, quality, and access.^25^ ADSW stakeholders identified high‐priority research interests (eg, policy education, health care access) and collaborated with methodological experts to develop questions that generated both quantitative and qualitative data. This led to the design of a 24‐item questionnaire, consisting of dichotomous responses and free‐text open‐ended responses, to measure DOD reproductive health policy knowledge and explore participants' reproductive health experiences (see Supporting Information: Appendix S1). Data were collected between December 2021 and June 2022 through an online digital platform. To maximize anonymity, no demographic data were collected.

Descriptive statistics were generated for items with dichotomous responses. The codebook was developed using the framework *trajectories of women's abortion‐related care* with deductive codes modified to reflect unique military variables based on previous research (eg, role of military vs civilian providers, Uniform Code of Military Justice vs state laws).^26^ The codebook was finalized by one coder and one qualitative methods expert after completing the coding for one‐fifth of each free‐text item responses. Qualitative data were organized using MAXQDA 2024 and analyzed using thematic analysis.^27^, ^28^ Data were coded independently by 3 coders who met on a weekly basis to discuss results, resolve discrepancies through consensus coding, and document changes.^27^ All coders had previously served or were currently serving in the military and had experience in qualitative methods. Data were merged using comparison joint displays to determine where quantitative and qualitative data converged and diverged.^24^

Our research team acknowledges and respects that servicemembers capable of pregnancy and birth have held multiple identities and have not always identified as women. Because we also recognize that women comprise a small portion of the military and often experience marginalization, we intentionally use *servicewomen* in this study to ensure the term is present in the literature about military personnel.

## RESULTS

### Systemic Lack of DOD Abortion Policy Knowledge

Nearly all participants (94%; n = 167) reported personally knowing at least one other ADSW who had sought abortion care while on active duty (see Table 1). More than one‐third (35%; n = 63) stated that they knew 4 or more ADSW who sought abortion care while on active duty. Despite this, many stated they lacked awareness about DOD abortion policies and how these policies might impact their own care. For example, 65% (n = 115) of participants did not know TRICARE covered the cost of abortion in cases of rape or incest. Most participants (77%; n = 137) erroneously believed they required their leadership's permission to have an abortion, and 87% (n = 155) did not know they were entitled to convalescent leave to recover after having either a covered or noncovered abortion. Most (80%; n = 142) felt they did not receive consistent reproductive health information or resources from their current MTF (see Table 1). Common qualitative themes from these data included (1) lack of awareness of abortion policies and (2) unreliable knowledge sources and stigma (see Table 2).

**Table 1:** Department of Defense Reproductive Health Policy Knowledge Among Servicewomen Who Self Report Obtaining an Abortion on Active Duty

Questions	Yes n (%)	No n (%)
**Personal knowledge of servicewomen who terminated a pregnancy**		
How many women do you personally know who have terminated a pregnancy while serving in the military?		
0	11 (6)	
1	38 (21)	
2	34 (19)	
3	32 (18)	
4+	63 (35)	
**Policy knowledge**		
Were you aware that Tricare will cover pregnancy termination in the case of rape, incest, or danger to the mother?	63 (35)	115 (65)
Do you know how to access Tricare‐covered emergency contraception at your current location?	47 (26)	131 (74)
Were you aware that pregnancy termination is not considered an elective procedure and, therefore, does not require commander's approval?	41 (23)	137 (77)
Were you aware that you are entitled to convalescent leave through your military medical provider after any pregnancy termination, regardless if the termination was covered by Tricare?	23 (13)	155 (87)
In your experience as a patient, have you received consistent reproductive health information/resources from military providers at your current MTF?	36 (20)	142 (80)
**Leave, travel, and finance**		
Did you take personal leave leading up to, to obtain, or following the termination?	94 (53)	84 (47)
Did you have to travel more than one hour to have access to health care associated with pregnancy termination?	82 (46)	96 (54)
Did you experience financial difficulty as a result of seeking a termination?	85 (48)	93 (52)
**Stigma**		
Do you believe you've experienced discrimination because of a termination of others’ belief you terminated a pregnancy?	55 (31)	123 (69)
Were you granted convalescent leave as a result of your termination?	29 (16)	149 (84)
**Mental health**		
Were you offered mental health services for postabortive care?	14 (8)	164 (92)
Did you seek mental health services prior to or after your termination?	40 (22)	139 (78)
Would you have benefited from mental health services for postabortive care?	41 (23)	137 (77)

Abbreviation: MTF, military treatment facilities.

**Table 2:** Themes and Representative Quotations

Theme	Representative Quotations
Systemic lack of knowledge	As a victim of sexual assault, I have had the very real worry of having to consider an abortion but was told by the doctor they were not sure if it would be covered by TRICARE and if it wasn't how much it would cost. (Participant 108) I don't know if TRICARE covers abortion for any^a^ reason. (Participant 37) I don't know if TRICARE does cover abortions and at what stage. (Participant 45) I am not aware that TRICARE covers abortions or infertility treatments. I paid out of pocket for my abortion. (Participant 95) I was unaware about convalescent leave for post operation. I used my own leave. (Participant 107) Abortion is not an option with military health care unless baby is deceased within the womb. (Participant 38)^a^
Leave, travel, and financial burdens	I took a single day of leave for a preprocedure appointment on a Tuesday, and I had to fight for that specific day because a senior leader was trying to say I was “mission essential.” I got my abortion done on a Saturday of a three‐day weekend and returned to work on Tuesday. (Participant 112) Had to travel to another city and spend the night there. (Participant 59) I drove to another state for a cheaper clinic and did it on a weekend so I could be back for my shift on Monday night. (Participant 38) This was a straining time in my life, recent divorce, recent PCS move, paying 2 mortgages and car loan. (Participant 149) It was not vastly difficult but did require altering my budget for the next 6 months. (Participant 76) The abortion pill was $700 alone. I don't know what I would have done if I didn't have a savings. (Participant 107)
Stigma and professional repercussions	My previous supervisor and current section chief are both distant now. Both are very religious. Additionally, one of the nurses at the OB clinic was very dismissive and refused to help with the process. (Participant 43) My supervisor and section leadership treated me differently when I returned to work. I had to prove to them why I needed to take leave and would be out, they didn't want me to take leave initially. (Participant 58) At the time, my leadership was not supportive and pulled me and the father into the commander's office to discuss. Since then, I have answered questions about my previous pregnancy at most OB and women's health appointments (Participant 156) Abortion care for unexpected/unwanted pregnancies has been stigmatized and is seen rarely at an MTF with the implications it may have on careers especially to those who work within the servicing MTF and other possible ramifications. (Participant 109) I was 23 and newly engaged when I found out I was pregnant. However, my now‐husband and I had just commissioned in the Air Force and had no chance of being stationed together for at least 2‐3 years. I was not ready to have a baby, especially on my own. I went to the Women's Health Clinic to discuss my options, where I was abruptly turned away at the front desk and told that no one was allowed to talk to me about this. This made me feel even more isolated as a brand‐new second lieutenant who just arrived to her first duty station. (Participant 117)
Lack of mental health support	I felt shunned by my provider when I informed her of my plans. I received little to no assistance or support after the termination. “No follow‐up or even the offer of a [behavioral health] consult.” (Participant 87) I was not encouraged to seek mental health support for grief or postpartum care which would have benefited me during this traumatic experience that I still struggle with today. (Participant 151) Military mental health services failed to take issues seriously and brushed me off, leading to thoughts of suicide. (Participant 6)

Abbreviations: MTF, military treatment facilities; PCS, Permanent Change of Station; OB, obstetrician.

a Example of misinformation.

#### Lack of Awareness of Abortion Policies

Participants identified the lack of accessible, accurate information on abortion policies and resources as a systemic issue. Many participants questioned why reproductive health and abortion policies were not addressed during training when other health care policies and benefits were. Participant 66 felt that both male and female servicemembers “should have this knowledge before they need it” and that it should be incorporated during basic and officer training. Other participants called for standardized training across the DOD, similar to other health‐related content that is presented during annual training:

We need to do better for [ADSW]. The resources are not consistent from base to base. The access to information isn't available (we don't know what we don't know), as a [military leader] this info is crucial to pass to my airmen and I'm not even as informed as I should be. (Participant 129)

Participants expressed frustration with the stigma surrounding abortion in the military, which they felt also contributed to the widespread lack of knowledge regarding abortion policies and resources. Participant 42, despite having had an abortion and served in multiple leadership roles, stated “abortion access is a taboo subject, and I have never heard anything about it … in my 12 years in the military.” Participant 127, a medical professional, expressed her dismay at the culture of silence, stating “the military has an outdated culture when it comes to abortions. I had no idea about the resources, and my spouse and I both work in the MTF.”

#### Unreliable Knowledge Sources and Stigma

Participants felt military leadership and military health care professionals provided little or poor‐quality information about covered and noncovered abortion policies and resources and often struggled to identify reliable knowledge sources. One expressed frustration:

There was a complete lack of guidance and understanding of options regarding termination from my command team to the local med group. Also a lack of online resources to help explain guide me through process. Aside from the small excerpt in [Air Force Instruction manual]—there was nothing further to help me understand options. (Participant 151)

This lack of information aggravated participants and exacerbated the many challenges they faced when attempting to make informed decisions. Participant 34 described being “immediately turned away and told [the MTF] couldn't even discuss options” while she was pregnant and trying to determine her next steps (ie, parenthood, adoption, abortion). Participant 60 was denied clinic assistance when trying to apply for convalescent leave and found military providers were “unable or unwilling (don't know which with my experience) to point us in the right direction” of identifying and accessing safe and legal abortion care.

Lack of knowledge, coupled with stigma, further compounded access to reliable and trustworthy information. Due to fear and stigma surrounding abortion care in the military, Participant 23 observed, “unless you know someone who has undergone the procedure, it is very difficult to get the information.” Participant 101 commented that there is “limited information on termination information. Not easy to get an appointment with women's health, and from my experience, the doctors are mostly male, which makes it that much harder to have this conversation.” This atmosphere creates barriers to care by discouraging ADSW from asking questions, with Participant 82 noting, “I didn't know about abortion support and I was too scared/embarrassed to ask.” To address ADSW's limited knowledge of abortion care policies, participants called for mandatory education during training and proactive dissemination of information during unit‐level training:

Every female serving should be given this information up front rather than waiting until they get to that stage of their life. Constant education, even if it's a section in the Patient Portal that lists all these resources and frequently asked questions, would greatly benefit service members. It's still very taboo and a lot of women/families end up grieving alone. (Participant 47)

### Lived Experience of Accessing Abortion Care

#### Travel and Financial Burdens

More than half (53%, n = 94) of participants had to take personal leave to seek abortion care. Overall, 46% (n = 82) traveled more than one hour to receive care, with 15% (n = 27) having to travel to another state or country to obtain safe and legal abortion care. Participants who paid out of pocket (66%, n = 117) reported costs ranging from $350 to $7000, with 48% (n = 85) stating that they experienced financial difficulties.

Because TRICARE does not cover abortion care except in cases of rape or incest or if the life is endangered, many participants described it as “an unexpected expense” (Participant 65). Even for those with savings, it was difficult to budget due to the variability in travel and health care costs, with Participant 140 stating, “the information I was given prior [about cost] was not accurate and the procedure ended up costing almost twice as much.” Those who did not experience financial difficulties described having “a good amount of money in savings already” (Participant 121) and frequently identified their higher military rank and higher salaries as a facilitator to care because they “could afford to spend as an officer” (Participant 79). Enlisted rank was a financial barrier due to lower income: “I was an E3 [junior enlisted] on birth control because I already had 2 children. It was very hard not to have that $800 that month” (Participant 165). Those experiencing financial hardship described having to “sell a car” (Participant 54) to raise the money or deciding to “either pay rent or terminate. It was a tough choice” (Participant 101).

#### Stigma and Negative Professional Repercussions

Although ADSW are permitted to legally receive abortion care, 31% (n = 55) of participants experienced negative professional repercussions in the workplace after having an abortion. This figure likely underrepresents the true extent of the issue, as many participants simply “didn't tell anyone” (Participant 132) or “outwardly labeled [the abortion] a miscarriage to avoid the judgment” (Participant 118) and potential associated repercussions. However, participants who disclosed their decision to obtain abortion care experienced a pattern of professional consequences affecting their career progression, job placement, and performance assessments. Participant 158 reported her “immediate supervisor who was extremely conservative lectured me about Jesus even though I am NOT Christian. He forced his religious beliefs on me” while Participant 6 was denied additional time off to physically recover “because [leadership] disagreed with [her] decision to have an abortion.” The stigma and professional repercussions also extended to military spouses. Participant 98 shared, “[my spouse] was being supportive of my decision and paid the consequences” by being professionally penalized by his unit leadership. Participant 84 reported that her Enlisted Performance Report [an annual job rating that influences promotion] “was influenced by it [abortion],” and that her decision to seek abortion care negatively affected her formal performance evaluation.

The fear of stigma and retaliation extended to interactions with health care providers. Participant 106 recalled how

The OB/GYN clinic and the female ER nurses were wonderful, but the male ER doctor was critically questioning me visiting Planned Parenthood and short with me the whole visit. I was scared he was going to report me, and he made me feel more guilty than I already was about seeking an abortion.

Many participants raised concerns about disclosing their abortions to military health care providers even years after receiving care. Participant 174 was “petrified to tell my gyn provider about it for fear I would be punished” although it is legal for ADSW to obtain abortion care. This culture of stigma, secrecy, and fear further compounds the challenges faced by ADSW, creating an environment in which many feel unable to seek the support and care they need without risking their careers and relationships within the military community.

#### Lack of Mental Health Support

Only 16% (n = 29) of participants were granted any form of convalescent leave to recover. The vast majority (92%, n = 164) were not offered postabortion mental health support, and 78% (n = 139) did not seek such services before or after their abortion, yet 77% (n = 137) of participants felt that they would have benefited from access to mental health care.

Mental health was identified as an influential factor at all points of the abortion care trajectory. Participant 103 stated their decision to have an elective termination was based on an “unstable relationship [with her partner] and [her own] mental health” concerns. Providers involved in the care process (eg, pregnancy identification, options counseling, dilation and curettage procedure, abortion) did not routinely offer to connect patients with mental health resources. This was experienced by participants who had covered abortions and those who had noncovered abortions. Participant 25, who required several medical appointments, reported “no doctor offered me mental health care,” although she told them she was struggling emotionally. Participants expressed a desire for military providers to be proactive in offering mental health services, with Participant 31 suggesting “the initial identification of pregnancy is an opportunity for a health care provider to discuss mental health and convalescent options for members if they opt not to carry the pregnancy to term.”

Participants’ concerns about disclosing abortion care influenced if and how they sought mental health support. In some cases, participants were required to “explain why [they] were seeking mental health in order to receive mental health care” (Participant 137), forcing them to disclose their abortion to additional personnel. Participant 49 decided not to speak with a mental health professional “due to the potential discrimination or feeling of inability to receive care or treatment,” and Participant 89 reported “the mental health care I sought after my abortion was not within the military clinic due to fear of consequences to my career.” The fear of forced disclosure to leadership, stigmatization, and professional repercussions around the decision to have an abortion acts as a barrier to seeking mental health care.

Participants who accessed mental health care were often already receiving mental health care prior to their abortion and “able to get an extra appointment” (Participant 25) with their treating provider. A lack of access to mental health services or empathetic providers led to long‐term effects for some participants. Participant 73 explained how “the mental health conditions I've suffered because of my traumatic abortion experience include depression, anxiety, and PTSD.” The desire for mental health care stemmed from participants coping with miscarriages or feeling isolated by their decision to have an abortion but not being able to discuss it with others due to stigma and fear of professional repercussions.

## DISCUSSION

### Main Findings

ADSW who have accessed abortion care have significant knowledge deficits regarding DOD policies governing access to and resources for abortion care. With 64% of participants aware of at least one other ADSW who has had an abortion, a strong need for training on this topic exists. However, participants identified a systemic lack of education for ADSW, leadership, and service members about policies and protocols to facilitate access to abortion care services. Although previous research identified knowledge deficits at the individual level,^16^, ^17^, ^20^, ^29^ our findings highlight the critical need to provide formal education on DOD policies to ADSW and leadership, especially given the dynamic sociopolitical environment for reproductive health care access. More than half of participants had to take personal leave to obtain abortion care, 46% had to travel more than an hour to reach a provider, and 48% reported that accessing abortion care caused financial difficulties. Our findings reflect previous research that has identified financial and logistical barriers for ADSW accessing care but provide clarity to the scope of the issue, which has previously gone unstudied.^9^, ^11^, ^23^ Consistent with previous research, ADSW seeking abortion care continue to experience stigma and professional repercussions associated with seeking care.^18^, ^19^, ^23^ Furthermore, our findings indicate that this stigma impacts not only their careers but also those of their spouse. Many participants disclosed they did not seek mental health care following their abortion due to concerns about privacy and stigma.

### Policy Recommendations

It is imperative that ADSW have universal access to health care, regardless of duty assignment or state. The DOD should develop and implement a standardized abortion policy and access training for military health care professionals, leadership, and servicemembers that is consistent with recommendations of professional organizations such as the American College of Obstetricians and Gynecologists.^30^ Such training would address the systemic knowledge deficits that contribute to delays in care. This training should incorporate material that destigmatizes the decision to seek abortion care and include resources for those who feel they have been unjustly penalized. To further reduce stigma and repercussions for ADSW, the DOD should mandate additional privacy protection for members regarding command notification of pregnancy, antidiscrimination measures to protect ADSW who have had an abortion, protections for military health care providers to practice at MTFs in accordance with federal guidelines, and legal protection for servicemembers in states with so‐called abortion *bounty‐hunter* laws that allow for the prosecution of those who aid or abet women seeking abortion care.^31^ Regarding increasing access to mental health services following abortion care, the DOD should explore policies facilitating the uptake of telehealth options to enhance patient privacy. Standardized education should ensure military providers are familiar with resources specific to postabortion mental health care support, such as Postpartum Support International's virtual support groups and the Maternal Mental Health Hotline.^32^, ^33^

Following the Dobbs decision, the DOD urgently needs to mandate policies that provide additional protections for the nearly 100,000 servicewomen who currently receive care in states with restrictive laws that reduce accessibility of care for patients in life‐threatening situations (eg, extraction of a servicemember in a civilian care setting for emergency transport to an MTF that is permitted to perform lifesaving care).^9^ This policy should be backed by a 24‐hour crisis line ready to dispatch support services in addition to legal counsel. With most large DOD training facilities and bases located in states with bans or restrictions on lifesaving abortion care, it is anticipated that almost all ADSW will be impacted by these laws at some point in their careers. Proactive DOD policy reform would contribute to the overall reproductive health and safety of servicemembers and their families stationed in states with such restrictions.

### Research Recommendations

This patient population has unique needs and considerations distinct from the civilian population, necessitating tailored research. Studies aimed at developing effective educational content regarding abortion access and DOD policies are critical to informing servicemembers and leadership about existing resources and for contributing to the destigmatization of abortion in the military. Furthermore, research on the broader implications of the Dobbs decision on military recruitment and retention is critical. This includes examining how state‐level abortion laws and restrictions may influence the willingness of servicemembers and potential recruits of reproductive age, both male and female, to serve in states with restrictive policies. Insights from such research are vital to understanding the potential effects on mission readiness and national defense. Research on abortion care via telehealth or online abortion medication providers should be performed to determine if such methods act as a facilitator to accessing abortion care and their impact. Investing in community‐based research in the military community that centers their voices and concerns is essential for improving policies, care, and reproductive health outcomes.

### Strengths and Limitations

To our knowledge, this is the first study examining the impact of abortion access on the mental health of ADSW. The importance of access to mental health care is critical in supporting the health and readiness of ADSW. In addition, although the use of snowball sampling can contribute to participant bias, this approach yielded a substantial qualitative sample (N = 178) from a historically difficult to reach population.

This study has several limitations. To protect participant anonymity and encourage honesty, demographic data were not collected, which may limit the generalizability of the findings. Additionally, all measures and military service details were self‐reported, which may limit the validity of the findings. Furthermore, unique visitors were not tracked, so it is not possible to know how many individuals did not complete the questionnaire.

## CONCLUSION

There is a systemic lack of policy knowledge among military health care providers, leadership, and ADSW. This stems from the absence of reliable, readily available information and resources for ADSW seeking abortion care, as well as the stigma surrounding abortion in military culture. ADSW are afraid to raise questions and concerns regarding abortion care and experience judgment and/or professional repercussions when disclosing their abortions. There is a deep desire for education and resources for ADSW before, during, and after abortion care. Ongoing policy development is essential to address these disparities and ensure that all ADSW, irrespective of where they are stationed, have access to comprehensive, high‐quality reproductive health care, which is critical for maintaining physical and mental health and mission readiness.

## CONFLICT OF INTEREST

The authors have no conflicts of interest to disclose.

## Supporting information




**Appendix S1**. Study Questionnaire

**Appendix S2**. Checklist for Reporting Results for Internet E‐Surveys (CHERRIES)

**Appendix S3**. Standards for Reporting Qualitative Research (SRQR) Checklist
