# Supplementary material for: Policy Knowledge and Abortion Access for US Active‐Duty Servicewomen: A Mixed‐Methods Study
Source: J Midwifery Womens Health. 2025 Nov 5;71(1):46–53. doi: 10.1111/jmwh.70049 (PMC12914618; doi:10.1111/jmwh.70049)
Supplement: Supplementary file 1 — Appendix S1. Study Questionnaire [file JMWH-71-46-s001.docx]

**Appendix S1. Study Questionnaire**

1. Do you believe Tricare covers adequate reproductive care for females? (e.g., birth control, infertility treatments, abortion access, OB care, etc.)
   1. Yes
   2. No

If you answered no to question #1, please specify: [Free text]

1. How many women do you personally know who have terminated a pregnancy while serving in the military?
   1. 0
   2. 1
   3. 2
   4. 3
   5. 4 or more
2. Of the women you know who terminated pregnancies while serving in the military, please check the main factor you believe influenced each of their decisions to terminate:
   1. N/A (I do not personally know any women who have terminated a pregnancy while in the military)
   2. Rape
   3. Incest
   4. Danger to mother
   5. Concerns regarding the health of the baby
   6. Unplanned pregnancy
   7. Other [free text]
3. Have you terminated a pregnancy while serving in the military?
   1. Yes
   2. No (Skips to Question #15)
4. What was your reason for terminating the pregnancy?
   1. Rape
   2. Incest
   3. Danger to mother
   4. Concerns regarding the health of the baby
   5. Unplanned pregnancy
   6. Other [free text]
5. Did you take personal leave leading up to, to obtain, or following the termination?
   1. No
   2. Yes

If you answered yes to question 6, please specify each leave and intent (e.g. to travel, to heal, to grieve): [Free text]

1. Did you have to travel more than one hour to have access to health care associated with pregnancy termination?
   1. No
   2. Yes

If you answered yes to question 7, please specify: [Free Text]

1. How much in out-of-pocket expenses did you incur as a result of travel and termination access? [Free Text]
2. Did you experience financial difficulty as a result of seeking a termination? Please explain. [Free Text]
3. Were you granted convalescent leave as a result of your termination?
   1. No
   2. Yes
4. Did you seek mental health services prior to or after your termination?
   1. No
   2. Yes
5. Were you offered mental health services for post-abortive care?
   1. No
   2. Yes
6. Would you have benefited from mental health services for post-abortive care?
   1. No
   2. Yes
7. Do you believe you’ve experienced discrimination because of a termination or others’ belief you terminated a pregnancy?
   1. No
   2. Yes

If you answered yes to question 14, please specify: [Free Text]

1. Before this feedback, were you aware that Tricare will cover pregnancy termination in the case of rape, incest, or danger to the mother?
   1. No
   2. Yes
2. Before this feedback, were you aware that Tricare will cover emergency contraception (e.g.: Plan B, Morning After Pill, etc.) without a prescription?
   1. No
   2. Yes
3. Do you know how to access Tricare-covered emergency contraception at your current location?
   1. No
   2. Yes
4. Before this feedback, were you aware that you are entitled to convalescent leave through your military medical provider after any pregnancy termination, regardless if the termination was covered by Tricare?
   1. No
   2. Yes
5. Before this feedback, were you aware that pregnancy termination is not considered an elective procedure and, therefore, does not require commander’s approval?
   1. No
   2. Yes
6. Have you experienced any of the following while pregnant in the military due to local OB policy affecting your care (check all that apply)
   1. N/A (have not been pregnant in the military)
   2. Illness when pregnant
   3. Traumatic birth
   4. Miscarriage
   5. Unidentified/late-identified pregnancy
   6. Unidentified/late-identified fetal abnormality
   7. Other [Free text]
7. In your experience as a patient, have you received consistent reproductive health information/resources from military providers at your current MTF?
   1. N/A (I have only been assigned at one location)
   2. Yes, it has been consistent (does not vary by provider) at my current MTF
   3. No, it has not been consistent (it varies by provider) at my current MTF
8. In your experience as a patient, have you received consistent reproductive healthcare resources at each duty station or state you’ve been assigned?
   1. N/A (I have only been assigned at one location)
   2. Yes, it has been consistent (does not vary by base or state) across my career
   3. No, it has not been consistent (it varies by base or state) across my career
9. Is there anything else you would like to share about your experience accessing birth control or abortion care? If so, please free-text in the below box:
