# Supplementary material for: Policy Knowledge and Abortion Access for US Active‐Duty Servicewomen: A Mixed‐Methods Study
Source: J Midwifery Womens Health. 2025 Nov 5;71(1):46–53. doi: 10.1111/jmwh.70049 (PMC12914618; doi:10.1111/jmwh.70049)
Supplement: Supplementary file 2 — Appendix S2. Checklist for Reporting Results for Internet E‐Surveys (CHERRIES) [file JMWH-71-46-s002.docx]

**Appendix S2. Checklist for Reporting Results for Internet E-Surveys (CHERRIES)**

| **Item Category** | **Checklist Category** | **Description/Location in Manuscript** |
| --- | --- | --- |
| Design | Describe Survey Design | Abstract; Methods |
| IRB (Institutional Review Board) approval and informed consent process | IRB Approval | Methods |
|  | Informed Consent | Methods |
|  | Data Protection | All data were stored on a research-secure server. Only members of the research team could access data. |
|  |  |  |
| Development and pre-testing | Development and testing | Methods |
| Recruitment process and description of the sample having access to the questionnaire | Open survey versus closed survey | Methods; The questionnaire in the study was open to those who received the link to the survey. |
|  | Contact mode | Methods |
|  | Advertising the survey | N/A |
|  |  |  |
| Survey administration | Web/E-mail | Methods |
|  | Context | Methods |
|  | Mandatory/Voluntary | Voluntary |
|  | Incentives | No incentives were offered for completing the questionnaire. |
|  | Time/Date | Methods |
|  | Randomization of items or questions | No, items and questions were not randomized. |
|  | Adaptive questioning | N/A |
|  | Number of items | Methods |
|  | Number of screens (pages) | 3 pages |
|  | Completeness check | 12 items required answers that were confirmed with java script check prior to submission; not all questions applied to all participants; not all participants wished to answer why they responded a certain way to a question. |
|  | Review step | N/A – entire questionnaire was accessible until participant submitted their answers. |
|  |  |  |
| Response Rates | Unique site visitors | N/A; Discussion: Strengths and Limitations |
|  | Vies rate (Ratio of unique survey visitors/unique site visitors) | N/A |
|  | Participation rate (Ratio of unique visitors who agreed to participate/unique first survey page visitors) | N/A |
|  | Completion rate (ratio of users who finished survey/users who agreed to participate) | N/A |
| Preventing multiple entries from the same individual | Cookies used | N/A |
|  | IP check | N/A |
|  | Log file analysis | N/A |
|  | Registration | N/A |
|  |  |  |
| Analysis | Handling of incomplete questionnaires | All quantitative data was complete as participants could not submit without answering those questions; qualitative data entry was voluntary due to the personal nature of the content, we did not consider the absence of qualitative data as incomplete data. |
|  | Questionnaires submitted with an atypical timestamp | N/A |
|  | Statistical correction | N/A |
